# The dynamics of vegetation diversity and biomass under traditional grazing in Ethiopia's Somali rangeland

**DOI:** 10.1002/pei3.10127

**Published:** 2023-11-07

**Authors:** Haftay Hailu Gebremedhn, Sintayehu Werkneh Dejene, Samuel Tuffa, Yayneshet Tesfay, Sylvanus Mensah, Adam John Mears Devenish

**Affiliations:** ^1^ African Center of Excellence for Climate‐Smart Agriculture and Biodiversity Conservation Haramaya University Dire Dawa Ethiopia; ^2^ Alliance of Biodiversity International and CIAT Dire Dawa Ethiopia; ^3^ Oromia Agricultural Research Institute Addis Ababa Ethiopia; ^4^ Independent Consultant Mekelle Tigray Ethiopia; ^5^ Laboratoire de Biomathématiques et d'Estimations Forestières, Faculté des Sciences Agronomiques Université d'Abomey Calavi Cotonou Benin; ^6^ Chair of Forest Growth and Dendroecology Albert‐Ludwigs‐Universität Freiburg Freiburg im Breisgau Germany; ^7^ Royal Botanic Gardens Surrey UK

**Keywords:** Ethiopia pastoralism, herbaceous species diversity, rangeland productivity, vegetation attributes

## Abstract

Traditional grazing management practices are central to rangeland productivity and biodiversity. However, the degradation of rangelands and loss of ecosystem services have raised concerns about the future of pastoralism as a form of land use. It is imperative to understand how these practices influence vegetation attributes, e.g., herbaceous species diversity and composition, growth forms (grass, forbs), life form (annuals, perennials), tree metrics (density, canopy cover, and biomass). This study evaluates vegetation shifts under three grazing management practices‐enclosures, open grazing, and browsing lands‐in the Somali pastoral ecosystem of Ethiopia. Enclosures exhibited the highest diversity in herbaceous species, with open grazing lands favoring forbs and annuals. Distinct compositional shifts in herbaceous species were observed across regimes, especially in grass and annuals. Enclosures had three times higher herbage biomass of open grazing and double that of browsing management practice. Conversely, browsing management practices presented optimal wood biomass, density, and canopy cover. The results highlight that a transition to combined enclosure and browsing practices can elevate plant production and diversity, benefiting the Somali rangeland economy. Consequently, dryland restoration should incorporate indigenous knowledge to ensure future rangeland sustainability and biodiversity preservation.

## Graphical abstract

This study explores the impact of different grazing management practices on vegetation attributes in the Somali pastoral ecosystem of Ethiopia. It examines herbaceous species diversity, composition, growth forms (grass vs. forbs), life forms (annuals vs. perennials), and tree metrics (density, canopy cover, and biomass) under three grazing regimes: enclosures, open grazing, and browsing lands. The findings reveal that enclosures promote the highest herbaceous species diversity, while open grazing areas favor forbs and annuals. Combining enclosure and browsing practices can enhance plant production and diversity, contributing to the sustainability of Somali rangelands and biodiversity preservation.

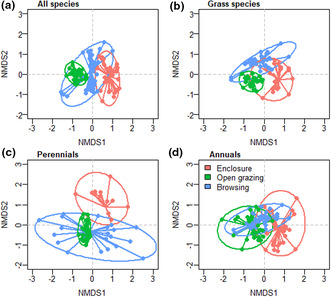

## 1 INTRODUCTION

Rangelands are a fundamental component of the global ecosystem, covering an estimated 54% of the world's terrestrial area (Teillard et al., 2016). These landscapes support a diverse array of plants, animals, and microbes, holding ecological, economic, and cultural importance (Seid et al., 2016). Furthermore, rangelands supply about 70% of the global forage to both domestic and wild ungulates through grazing and browsing (Derner et al., 2006). In Africa and Ethiopia, rangelands' cover 43% and 62% of the total landmass respectively. They serve as the primary source of feed for ruminants and crucial habitat for dry‐land biodiversity. However, challenges like poor management, population growth, deforestation, overgrazing, and changing land use and climate patterns have degraded these arid and semi‐arid ecosystem (Maitima et al., 2004; Miehe et al., 2010; Oba & Kotile, 2001). Factors exacerbating this degradation include the loss of traditional indigenous knowledge and reduced elder participation in rangeland management (CARE, 2015; Oba, 2012). This has led to a decline in rangeland resources and biodiversity, adversely affecting forage production and conservation efforts.

Traditional grazing management practices significantly influence the vegetation functional and structural attributes of rangelands. For instance, implementing grazing enclosures in dryland areas has been shown to enhance vegetation productivity (Pucheta et al., 1998) and promote species diversity (Abebe et al., 2006; Hailu, 2017). However, Tagesson et al. (2016) noted that areas with higher grazing intensity displayed superior vegetation productivity compared to low‐grazing areas. Multiple studies have highlighted that a mobile livestock grazing strategy supports biodiversity conservation and sustainable rangeland management (Angassa & Beyene, 2003; Tefera et al., 2007a; Treydte et al., 2011). In contrast, areas with restricted livestock mobility risk overgrazing, which can undermine biodiversity and biomass yields.

Identifying species that are essential to rangeland ecosystem functioning is challenging due to intricate interactions. In response, applied ecologists have recommended classifications based on functional groups (Chieppa et al., 2020; Feagin & Wu, 2007; Hashemi et al., 2018; Pettit et al., 1995), categorizing rangeland plants by phenology, growth form (grass vs. forbs), and reproductive strategy (annuals vs. perennials). Distinct responses to grazing between forbs and grasses have been observed regarding their vegetation cover (Fulbright et al., 2021; Stahlheber & D'Antonio, 2013). Moreover, species responses to grazing vary with their life form and reproductive strategy, with annuals often being more disturbance‐tolerant than perennials due to faster growth rates (McIntyre & Lavorel, 2001). However, studies on the plant functional group's response to traditional grazing are limited in the East African rangelands. Most studies have traditionally focused on maximizing forage productivity rather than considering plant diversity. This approach may result in misinterpretation regarding local species diversity conservation, restoration, and management of these rangelands. Therefore, studying the response of plant functional groups to traditional grazing is crucial for a comprehensive understanding of the overall ecological dynamics.

The Somali Regional State in Ethiopia is primarily a pastoral ecosystem, with rangelands comprising 90% of its area (Gezahegn, 2006). Pastoralists in this region use various traditional rangeland resource management strategies, including enclosures and herd‐splitting, to enhance vegetation recovery and adapt to drought and variable ecosystem productivity. For instance, they divide herds into browsers and graziers to address the uneven distribution of rangeland resources. The potential benefits of these alternative management practices have often been overlooked by extension and research services, especially knowledge on the impacts of various indigenous grazing management practices on rangeland vegetation functional and structural attributes including plants functional groups across growth (grass and forbs) and life forms (annuals and perennials).

This study evaluates the impacts of three grazing management regimes—enclosure, open grazing, and browsing lands—on herbaceous and woody species composition, diversity, canopy cover, and biomass in the Somali Region of Ethiopia. We hypothesis that transitioning from deeply ingrained traditional open grazing, often linked with vegetation attribute decline, to contemporary indigenous grazing methods can boost vegetation productivity. We further hypothesize that these adaptive grazing strategies, evolved in response to dwindling rangeland resources, are instrumental in preserving vegetation functional (species diversity and composition) and structural (density and biomass) attributes.

## 2 MATERIALS AND METHODS

### 2.1 Description of the study area

**FIGURE 1:**
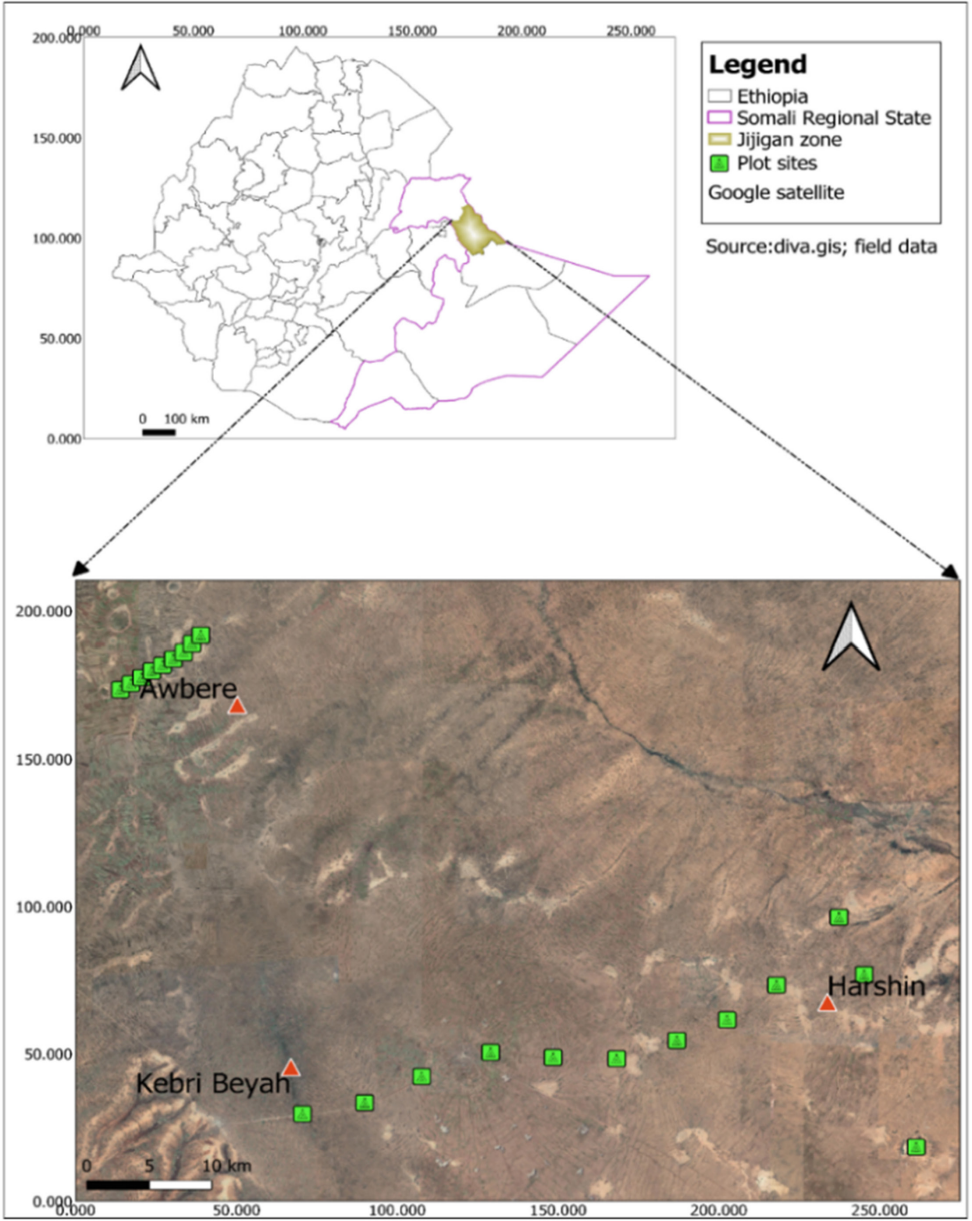
Location of the study area and experimental site layout.

The study was conducted in the Jigjiga field site (9.35° N, 42.79° W) located in the Somali Regional State of Ethiopia (Figure 1), a part of the expansive East African pastoral ecosystem. The area experiences two rainy seasons annually: the main season from July to September and a shorter season from March to April. With an average annual rainfall of 660 mm (Gebremedhn et al., 2022), the region predominantly features sandy loam soils (Alemie & Gebremedhin, 2019). Temperatures remain high year‐round, averaging between 20°C and 35°C (Gezahegn, 2006). The local vegetation consists of wooded grasslands dominated by species such as *Acacia etbaica, A. busse, Vachellia nilotica, Chrysopogon aucheri*, and *Eragrostis* spp (Hailu, 2017). The predominant land‐use system is pastoralism, with the community relying heavily on livestock and utilizing much of the land for grazing.

### 2.2 Site selection

A reconnaissance survey was conducted with the help of local natural resource management experts and elders who had considerable knowledge of grazing management practices in the study area. This enabled us to identify and choose three dominant and traditional grazing management practices for this study: enclosures, communal open grazing, and browsing land. Enclosures are part of the rangeland that remains fenced off until sufficiently rehabilitated and ready for hay production. They are often used on the basis of scheduled cuts and carry when there is a feed shortage for grazing in the open communal grazing areas. Communal open grazing areas are characterized by open grass vegetation with scattered trees and are used for extensive livestock grazing throughout the year. The communal open grazing area represents the traditional and most common land‐use system in the Somali rangelands. This form of land use is based on the principles of collective actions whereby community members have equal access to grazing and water resources throughout the year. Browsing lands are a responsive grazing system designed to adapt to changing environmental conditions and enhance resilience to climate change. It involves the deliberate division of herds based on their diversity and an understanding of local ecosystems. Pastoralists categorize grazing areas into subcategories that are based on factors such as plant cover, soil types, and ecological knowledge. Herd diversity plays a crucial role in optimizing resource utilization since different domestic animals, such as camels and goats, are browsers, while sheep and cattle are grazers. This approach maximizes the use of available fodder. One specific subcategory, referred to as “bay land,” features open bush mixed vegetation and is utilized for camel and goat browsing when feed becomes scarce in the grass‐dominated communal open grazing areas.

The three grazing management practices are implemented within the Jigjiga zone of the Somali regional state. For the purpose of this study, these practices were considered in areas with similar climate, soil, and ecological characteristics (see Figure 1). The only notable difference among them is the way local pastoralists utilized the grazing land resources, including the intensity, frequency, and timing of harvesting grass and browsing materials. For instance, in the open grazing systems, various livestock species such as cattle, goats, sheep, and camels grazed the area throughout the year. During the vegetation growth period and early dry season, there is an abundance of forage biomass with good nutritive value. However, as the dry season progresses, forage biomass decreases, prompting many pastoralists to relocate their herds in search of better feeding resources, dividing their herds according to suitable grazing areas. Goats and camels are typically allocated to areas dominated by bushes (browsing lands). These browsing lands remain accessible throughout the year, but grazing intensity decreases during the rainy season when there is sufficient forage biomass available in the communal open grazing areas. Enclosure areas are reserved for herds consisting of milking cows, sick animals, and calves that are unable to travel long distances in search of feed and water.

### 2.3 Sampling design and data collection

A transect survey method was used to sample vegetation attributes across the three different management practices. Specifically, nine square plots of 400 m^2^ each were established at an interval of 5 km for the open grazing practice along the Harishin and Kebri Beyah rangelands (approximately a distance of 45 km) of the Jigjiga zone (Figure 1). Similarly, nine 400 m^2^ plots were laid at an interval of 1 km for the browsing land management practice in Awebere rangelands of the same zone (Figure 1). We randomly selected three private enclosures of an average size of 1–2 ha each, in which three 400 m^2^ plots at an interval of 30 m were established, also yielding nine plots for vegetation sampling.

We measured tree/shrub density, canopy diameter, canopy height, and stem height of woody species in each 400 m^2^ plot. Canopy cover was calculated using the average of the two longest canopy diameters perpendicular to each other and parallel to the ground, following the method of Greig‐ Smith (1983). Stem height was measured as the total height of the plant stems from the ground level to the highest foliage. For species with multiple stems, each stem was measured separately, and the average value was calculated. Height measurement and canopy length and width were conducted for the whole plant by measuring multiple stems as if it were one tree.

Herbaceous vegetation attributes (e.g., species names, abundance, and biomass) were measured in five sub‐quadrats of 1 m^2^ placed within each of the 400 m^2^ plots following a zigzag pattern (i.e., four at all corners and one at the middle position). In total, 135 quadrats were laid out for sampling herbaceous vegetation. Species richness was recorded as the sum of all herbaceous species present in the 1 m^2^ quadrats. The nomenclature of the plant species followed the Flora of Ethiopia (Hedberg et al., 2003). The recorded species were categorized into three desirability classes based on their preference for grazing by livestock animals, using local ecological knowledge derived from herders and documented literature (Jerry et al., 1989). In addition, we identified all herbaceous vegetation within the quadrats as either grass or non‐grass species (forbs) following Behnke (1986). We measured aboveground herb biomass by harvesting live and dead material inside each 1 m^2^ quadrat at ground level. We weighed the harvested samples in the field to obtain fresh weight. Thirty percent of the harvested samples from each quadrat were placed in a paper bag for dry matter analysis.

### 2.4 Data processing and laboratory analyses

For woody aboveground biomass estimations relied on the allometric biomass equations as propounded by Hasen‐Yusuf et al. (2013). Herb samples harvested from the field were oven‐dried at a temperature of 105°C over 48 h, post which their dry weight was determined. The cumulative dry biomass for each quadrat was derived by juxtaposing the proportion of each dried sample biomass to the comprehensive fresh biomass weight.

### 2.5 Data analyses

Statistical analyses were performed using the R Statistical Software version 4.1.1. (R Core Team, 2020). The impact of traditional grazing management practices on species diversity and composition was gauged via species accumulation curves and intricate multivariate analyses. First, species accumulation curves were constructed for all species as well as growth (forb and grass) and life (annual and perennial) forms based on the species x quadrats abundance matrix, with the help of the *accuncomp* function of the *BiodiversityR* package (Kindt & Coe, 2005). A sample‐based rarefaction procedure was used to estimate the 95% confidence intervals and compare the patterns of plant richness among traditional grazing management practices.

Species frequency collected in the plots was averaged for each of the three grazing management practices to determine the relative dominance of each species. To explore the impact of traditional grazing management practices on herbaceous species composition, we performed separate non‐metric multidimensional scaling (NMDS) for all species, as well as for growth and life forms. The NMDS was used to group plots with similar species into separate classes using the Bray–Curtis dissimilarity matrix. The NMDS analysis was performed using the *metaMDS* function in the *vegan* package (Oksanen et al., 2022). In addition, we performed correspondence analysis (CA) on the matrix of species abundance (frequency) per management practice with the FactoMineR package (Luo et al., 2009). The CA method uses a simple Chi‐square statistic to test for significant dependence between herbaceous species and management practices.

We used general linear models (GLMs) to test for significance (*p* < .05) effects of grazing management practices on canopy cover, tree density, and aboveground woody biomass. Specifically, binomial GLM was used for canopy cover modeled as percentage data (Zuur et al., 2009) whereas the Gaussian family was applied for tree density and aboveground woody biomass after log‐transformation. For herb biomass, we used a linear mixed‐effect with a quadrat nested within the plot as a random factor and management practice as the fixed factor. In case of significant effects of grazing management practices, group‐level averaged values were graphically presented for better visualization.

## 3 RESULTS

### 3.1 Herbaceous species richness and composition

**FIGURE 2:**
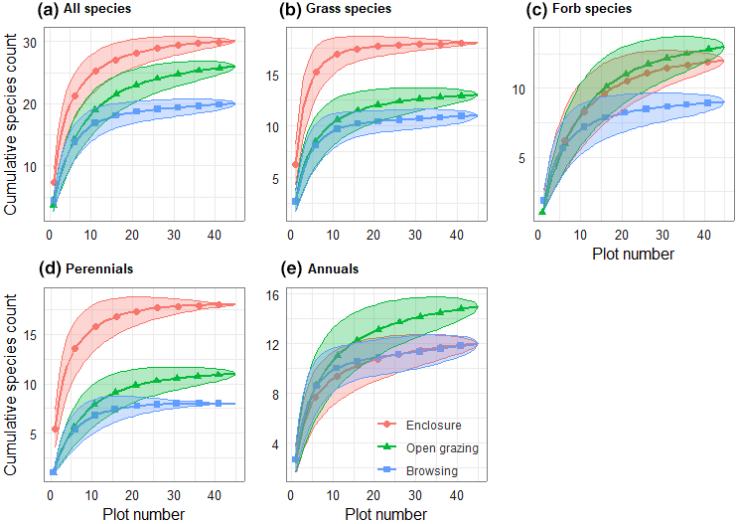
Species accumulation curves for all species and separately for growth and life forms in the 1 m^2^ plots.

A total of 47 herbaceous species were recorded (Table S1). The species accumulation curves for all species showed that enclosures were the most diverse management practice, followed by open grazing lands and browsing areas, which had the lowest species richness (Figure 2). Similar patterns were noted for grass species and perennials. Notably, forbs and annuals exhibited the most diversity in open grazing lands (Figure 2).

**FIGURE 3:**
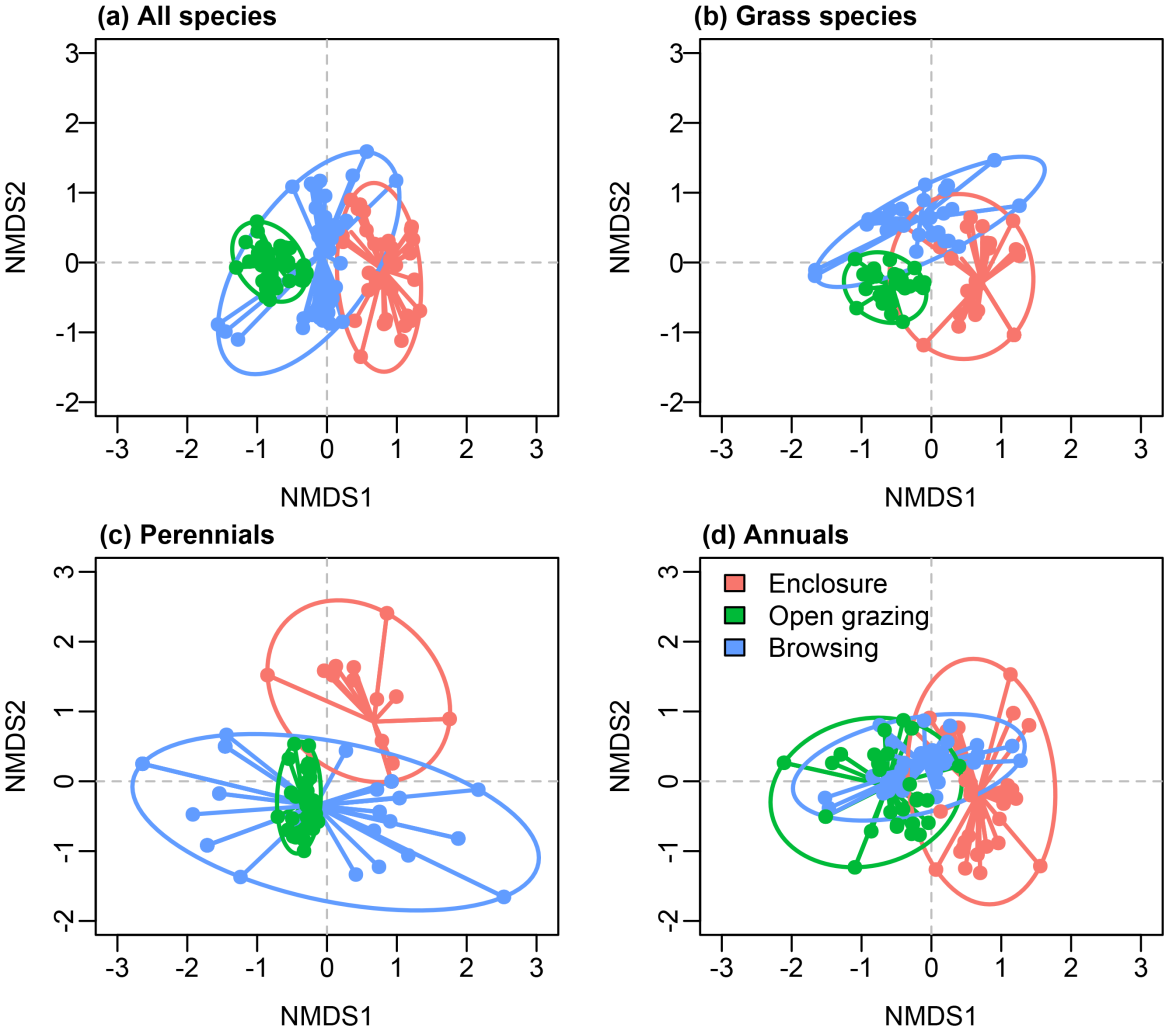
Ordination plot of plant community composition from non‐metric multidimensional scaling analysis (NMDS) for all species and separately for growth and life forms. Note that an ordination solution could not be reached for forbs because of insufficient data.

Species composition among different grazing management practices indicated a discernible shift (Figure 3a; Figure S1a). This shift was more evident in grass and annuals compared to perennials (Figure 3b–d; Figure S1b–d). Unfortunately, an ordination solution wasn't achievable for forbs due to insufficient data.

**FIGURE 4:**
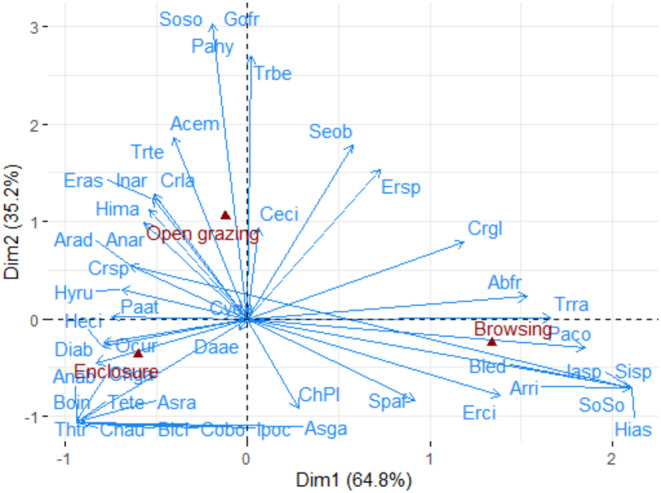
Asymmetric plot showing the distribution patterns of all herbaceous species according to enclosure, open grazing, and browsing management systems. Enclosure and browsing lands are associated with axis 1, whereas open grazing is associated with axis 2. Abbreviations for the species: Abfr, Abutilon fruticasum; Acem, Acanthus eminens; Anar, Anagallis arvensis; Anab, Andropogon abyssinicus; Aead, Aristida adoensis; Arri, Aristida rigida; Asra, Asparagus racemosus; Asga, Asystasia gangetica; Blci, Blepharis ciliaris; Bled, Blepharis edulis; Boin, Bothriochloa insulpta; Ceci, Cenchrus ciliaris; Chga, Chloris gayana; Chau, Chrysopogon aucheri; Chpl, Chrysopogon plumulosus; Crla, Crotalaria laburnifolia; Crsp, Crotalaria sp; Cyda, Cynodon dactylon; Daae, Dactyloctenium aegyptium; Diab, Digitaria abyssinicum; Ersp, Eragrostis sp; Erci, Eragrostis cilianensis; Eras, Eragrostis aspera; Gofr, Gomphocarpus fruticosus; Heci, Heliotropium cinerascens; Hias, Hibiscus asperhook; Hima, Hibiscus macranthus; Hyru, Hyparrhenia rufa; Inar, Indigofera arrecta, Ipoc, Ipomoea ochracea; lasp, lactuca sp; Ocur, Ocimum urticifolium; Paat, Panicum atrosanguineum; Pahy, Parthenium hystrophorus; Seob, Senna obtusifolia; Sisp, Sida sp; Soso, Solanum somalense; Spaf, Sporobolus africanus; Tete, Tetrapogon tenellus; Thtr, Themeda triandera; Trbe, Tragus berteronianus; Trra, Tragus racemosus; Trte, Tribulus terrestris.

The CA revealed a significant association between species and grazing management practices (Chi^2^ = 814; *p* < .001). The first axis predominantly represented browsing and enclosure (64.8%), while the second axis encapsulated open grazing areas (35.2%). The asymmetric plot illustrated a clear differentiation between the three grazing management practices and their associated herbaceous species. For instance, species like *Chrysopogon aucheri, Chlor*is *gayana, Digitaria abyssinicum*, and *Themeda triandera* species were widely distributed in the enclosure management sites (Figure 4). Conversely *Tragus berteronianus* and *Eragrostis* spp. species were highly distributed in the open grazing sites. In contrast, *Abutilon fruticosum, Hibiscus asperhook*, and *Tragus racemosus* species were dominant in the browsing sites (Figure 4).

The distribution of desirable herbaceous species was greater in the enclosure sites than in other management sites (Table S1). Among herbaceous species, desirable species of *Chloris gayana, Chrysopogon aucheri, Cynodon dactylon, and Themeda triandera* were distributed by 88.9%, 100%, 64.00%, and 93.33% respectively in the enclosure sites. In contrast, the distribution of undesirable herbaceous species was higher in the open communal grazing and browsing sites than in the enclosure sites. In the browsing site, the less desirable species of *Tragus racemosus and Sida sp* were highly distributed by 56.67% and 46.67%, respectively.

### 3.2 Wood species richness, dominance, density, and canopy cover

**FIGURE 5:**
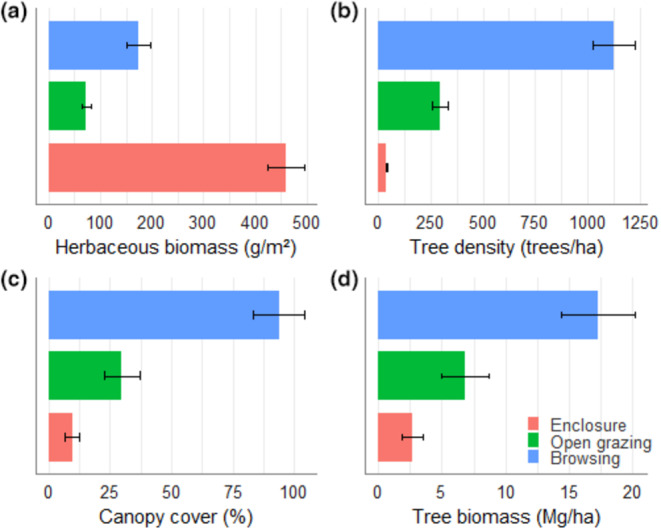
Bar plots showing means ± SE (standard error) of herbaceous biomass (a), tree density (b), canopy cover (c), and tree biomass (d) in an enclosure, open grazing, and browsing land.

Our survey enumerated five *Acacia* spp and one *Vachellia* spp. *Acacia etbaica* emerged as the dominant species in browsing sites, whereas *Acacia busie* prevailed in both the enclosure and open grazing sites (Table S2). Both tree density and canopy cover showed significant variations (*p* < .001; Table 1) across the different grazing management practices. Browsing sites exhibited superior woody density and canopy cover, followed by open grazing lands, with enclosures logging the lowest values (Figure 5).

**TABLE 1:** Effects of grazing management practices on herb biomass, tree density, canopy cover, and tree biomass.

	Df	Sum Sq	Mean Sq	*F*‐value/Chisq	*p*‐value
Herb biomass					
Management practice	2	69.47	34.74	63.64	3.10e‐10
Tree density					
Management practice	2	52.22	26.11	192.1	1.71e‐15
Residuals	24	3.26	0.14		
Tree canopy cover					
Management practice	2	—	—	68.9	1.08e‐15
Residuals					
Tree biomass					
Management practice	2	29.17	14.59	7.51	.003
Residuals	24	46.63	1.94		

Abbreviations: Chisq, Chi‐square; Df, degree of freedom; Mean Sq, mean square; Sum Sq, sum of squares.

### 3.3 Herb and woody biomass, and relation with diversity across grazing management practices

**FIGURE 6:**
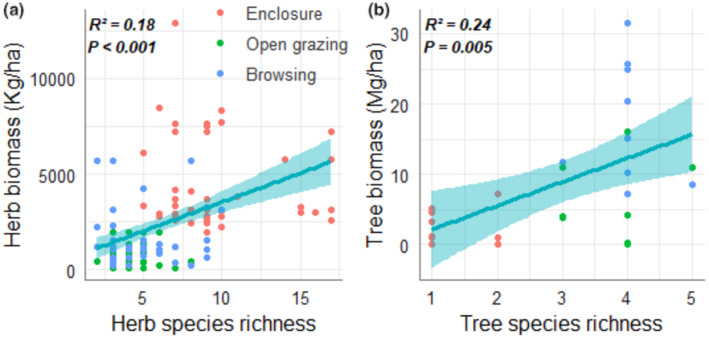
Scatterplots showing the relationship between species diversity and biomass for herbaceous (a) and trees (b) species separately.

Enclosure sites manifested a significantly higher herb biomass (GLM: *F* = 63.64; *p* < .001) than the other grazing management systems (Table 1; Figure 5). In contrast, browsing sites displayed significantly increased woody biomass (GLM: *F* = 7.51, *p* = .003) over open grazing and enclosure sites (Table 1; Figure 5). Moreover, we observed a positive correlation between species richness and biomass for both herbs and trees across the three grazing management practices (Figure 6).

## 4 DISCUSSION

Our research reveals marked variations in crucial vegetation attributes across the three grazing management practices employed by Somali pastoralists in Eastern Ethiopia. In the ensuing discussion, we delineate the ramifications of these practices on herbaceous and woody vegetation structures, as well as biomass. Our overarching aim is to underscore the implications of different strategies on rangeland health, productivity, and biodiversity preservation.

### 4.1 Species richness and composition

We found that herbaceous species accumulation estimates for enclosures, open grazing, and browsing areas were 30, 24, and 20, respectively (Table S1; Figure 2). A higher number of grass species and perennials were found in the enclosure sites while forbs species and annuals prevailed in the open grazing management sites (Figure 2). The sites considered as browsing management in this study declined in species richness possibly due to the presence of highly competitive tree densities. These results are consistent with prior research on tree‐grass interactions (Angassa et al., 2010; Hailu, 2016; Mekuria et al., 2018; Yayneshet et al., 2009), which demonstrated the efficacy of enclosure management in improving rangeland vegetation.

Moreover, grazing management practices had a significant impact on the segregation of species functional group composition of the rangeland (Table 1; Figure 3). The enclosure sites were dominated by grass species composition, whereas the communal open grazing sites had a dominant forbs species composition. Fulbright et al. (2021) noted that cattle selectively forage on grasses, which can reduce competition between forbs and grasses, resulting in an increased abundance of forbs. This suggests that certain forb species benefit from reduced competition with grass and were able to colonize the grazed areas. The current study finding of a higher proportion of perennial herbaceous species in enclosure management sites (see Figure 3) aligns with Pettit et al. (1995), who reported that perennial species dominated lightly grazed areas, while annual forbs and weedy species prevailed in heavily grazed locations. The dominance of annual species in the open grazing site might be due to their ability to complete their life cycle before summer, thus avoiding the season of highest grazing pressure.

Furthermore, the prevalence of preferred herbaceous species, such as *Chloris gayana, Chrysopogon aucheri, Cynodon dactylon, and Themeda triandera*, and *Themeda triandera*, was higher in enclosures than in other management sites (Table S1; Figure 4). The decrease in these desirable species under grazing and browsing management practices might be due to overgrazing. Gemedo‐Dalle et al. (2006) suggested that alterations in community composition resulting from grazing pressure could signal rangeland deterioration. Supporting this hypothesis, our study found a greater distribution of undesirable herbaceous species in grazing and browsing areas compared to enclosures. Similarly, Miehe et al. (2010) observed a marked increase in low‐quality herbaceous plants at a grazed site relative to a non‐grazed site in Senegal's savannah ecosystem.

Overall, our study provides evidence that the establishment of enclosures in response to declining rangeland resources is an appropriate management strategy in terms of maintaining herbaceous species diversity and composition. Therefore, it is essential to recognize the participation of pastoral communities in environmental monitoring and decision‐making as a fundamental aspect of effective rangeland management. Their knowledge and its outcomes can significantly contribute to the development of local policies (Oba, 2012).

### 4.2 Woody species density and canopy cover

The average tree densities per hectare in browsing, open grazing, and enclosure areas were estimated at 1125, 294, and 39, respectively. Browsing sites promoted higher tree density but still lower than the 2500 trees equivalent, which is the minimum threshold limit for woody species to be considered as threats for bush encroachment (Richter et al. (2001). Somali pastoralists view this increase in tree density as indicative of rangeland degradation, resulting in an imbalance in the grass‐to‐bush ratio, and consequently, a decrease in palatable herbaceous species diversity and productivity. Supporting this notion, the low number of herbaceous species richness in browsing management practices in our study may be due to woody vegetation encroachment. The phenomenon we observed in the browsing sites was consistent with other studies that demonstrated a shift in favor of trees and shrubs across many East African rangelands (Angassa & Oba, 2008; Gilo & Kelkay, 2017; Gobelle & Gure, 2018; Tefera et al., 2007a, 2007b; Woods et al., 2019; Yusuf et al., 2011).

In the current study, the density and canopy cover of woody species in the enclosure and open grazing areas were too small to be classified as a woody‐encroached state that significantly suppresses understory herbaceous species. However, the canopy cover in browsing land management practices exceeded the upper limit set at 90% beyond which the grazing value of rangeland is greatly reduced (O'Rourke and van Wijngaarden (1987). We speculate that the long‐standing practice of Somali pastoralists to split their herds as graziers and browsers reflects such dynamism in woody species density and cover noticed in the browsing sites. Consequently, policymakers and extension workers should acknowledge and recognize the Somali pastoralists' experiences and knowledge in maintaining browsing sites as important feeding niches for their livestock.

### 4.3 Vegetation biomass

The herbage biomass within enclosures was three times greater than that of open grazing areas and twice as much as in browsing land management practices (Figure 5; Table S3). This increase in herbage biomass in the enclosure management sites may be attributed to the overall better management inputs, including controlled grazing pressure. Consistent with our findings, studies conducted in Borana and other pastoral regions in Ethiopia have confirmed that periodically resting grazing lands enable herbaceous forage species to regenerate from the soil seed bank, thus increasing biomass yield (Abdulatife Ibrahim, 2016; Abebe et al., 2006; Angassa et al., 2010; Behnke, 1986; Mohammed et al., 2017; Nyberg et al., 2019). In contrast, the woody biomass in enclosures was approximately half that of open grazing areas and five times less than browsing land management practices (Figure 5; Table S3). This is attributable to the fit‐for‐purpose management style of enclosures by Somali pastoralists that of maximizing grass and hay production while maintaining minimum tree and shrub cover for shade. Such resilience of the local community is not given the level of support it needs to promote sustainable grazing systems and is often undermined by pastoralism policymakers. Finally, we found that, for herbs and trees, biomass was positively related to species richness (Figure 6). These positive effects of diversity on biomass are reminiscent of previous findings, and have repeatedly been explained by ecological mechanisms such as complementarity and dominance effects, which increase as herb/tree diversity also increases (Mensah et al., 2021; Noulèkoun et al., 2021).

## 5 CONCLUSION

Our study demonstrates that transitioning from open grazing to enclosure and browsing management practices can enhance plant production and foster better forage establishment in the Somali rangelands of Ethiopia. Specifically, we found that enclosures exhibit greater herbaceous species richness, abundance of desirable species, and biomass in comparison to open grazing and browsing management sites. Conversely, browsing areas exhibited significantly higher woody biomass, density, and canopy cover than the other grazing management practices. As a result, we advocate for the promotion of traditional pastoralist knowledge and practices, which entail splitting herds and assigning browser animals (camels and goats) to bush‐rich areas and grazers (cattle and sheep) to open grazing areas. Embracing traditional enclosure grazing management practices is essential for maintaining the resilience capacity of the natural environment and preserving rangeland ecosystem biodiversity. Consequently, rangeland restoration approaches should incorporate indigenous rangeland management practices to ensure the sustainable utilization of rangeland resources while upholding the social and cultural values of the community.

## CONFLICT OF INTEREST STATEMENT

The authors declare no conflicts of interest.

## Supporting information


Data S1:

## Data Availability

The data that support the findings in this study are available from the corresponding author upon reasonable request.
