# Supplementary material for: The dynamics of vegetation diversity and biomass under traditional grazing in Ethiopia's Somali rangeland
Source: Plant Environ Interact. 2023 Nov 7;4(6):342–52. doi: 10.1002/pei3.10127 (PMC10711641; doi:10.1002/pei3.10127)
Supplement: Supplementary file 1 — Data S1: [file PEI3-4-342-s001.pdf]

Supporting Information

Table S1. Abundance (%) of herbaceous species under different grazing management systems in the Somali Rangeland of eastern Ethiopia

Species name	Management practices			Growth form	Life form	Species Desirability
	Enclosure	Open Grazing	Browsing			
Abutilon fruticasum	-	8.89	26.66	F	A	UD
Acanthuseminens	4.44	11.11	-	F	P	UD
Anagallis arvensis	2.22	2.22	-	F	A	UD
Andropogon abyssinicus	28.89	-	-	G	P	D
Aristida adoensis	44.44	28.88	-	G	A	D
Aristida rigida	-	-	20.00	G	A	D
Asparagus racemosus	15.56	-	-	F	P	UD
Asystasia gangetica	35.56	-	-	F	P	LD
Blepharis ciliaris	8.89	-	-	F	P	LD
Blepharis edulis	-	-	20.00	F	P	LD
Bothriochloa insulpta	33.33	-	-	G	P	LD
Cenchrus ciliaris	46.67	68.88	31.11	G	A	D
Chloris gayana	88.89	-	-	G	P	D
Chrysopogon aucheri	100.00	-	-	G	P	D
Chrysopogon plumulosus	20.00	-	13.33	G	P	D
Conyza boranensis	8.89	-	-	F	P	D
Crotalaria glandifolia	-	4.44	6.67	F	P	D
Crotalaria laburnifolia	6.67	8.89	-	F	P	D
Crotalaria sp	-	-	8.89	F	P	D
Cynodondactylon	64.44	28.89	33.33	G	P	D
Dactyloctenium aegyptium	40.00	15.56	17.78	G	A	D
Digitaria abyssinicum	51.11	8.89	-	G	P	D
Eragrostis sp	-	33.33	22.22	G	A	D

Eragrostis cilianensis	17.78	-	57.77	G	A	D
Eragrostis aspera	40.00	51.11	-	G	A	D
Gomphocarpus fruticosus	-	11.11	-	F	P	UD
Heliotropium cinerascens	37.78	8.88	-	F	P	LD
Hibiscus asperhook	-	-	15.56	F	P	LD
Hibiscus macranthus	15.56	17.77	-	F	P	LD
Hyparrhenia rufa	40.00	20.00	-	G	P	D
Indigofera arrecta	15.56	20.00	-	F	A	LD
Ipomoea ochracea	8.89	-	-	F	A	LD
Lactuca sp	-	-	22.22	F	A	LD
Ocimum urticifolium	17.78	4.44	-	G	A	D
Panicum coloratum	-	2.22	17.788	G	A	D
Panicum atrosanguineum	31.11	11.11	-	G	A	D
Parthenium hystrophorus	-	2.22	-	F	P	UD
Senna obtusifolia	-	4.44	2.22	F	A	D
Sida sp	-	-	46.67	F	A	UD
Sporobolus africanus	15.56	-	24.44	G	P	D
Tetrapogon tenellus	4.44	-	-	G	A	LD
Themeda triandera	93.33	-	-	G	P	D
Tragus berteronianus	-	22.22	2.22	G	A	LD
Tragus racemosus	-	11.11	56.67	G	A	LD
Tribulus terrestris	4.44	11.12	-	F	A	LD

NB. F = forb, G = grass; A = annual, P = perennial; D = desirable species, LD = less-desirable species; UD = undesirable species

Table S2. Abundance (%) of wood species under different grazing management in the Somali Rangeland of eastern Ethiopia

**Species name**	**Management practices**		
	**Enclosure**	**Open grazing**	**Browsing (Bay)**
Acacia abyssinica	0	3.81	7.64
Acacia busie	50	40.95	10.59
Acacia etbaica	12.5	9.52	64.53
Acacia mellifera	12.5	10.48	6.40
Acacia toritilis	0	0.95	8.62
Vachellia nilotica	25	34.29	2.22

Table S3. Variation (mean ± standard error) of vegetation attributes between three grazing management systems in the Somali rangeland ecosystem of Ethiopia

**Parameters**	**Management practices**			
	**Enclosure**	**Open grazing**	**Browsing**	*P value*
Tree density (tree/ha)	39 ± 4.39[c]	294 ±39[b]	1125 ± 101[a]	<0.001
Canopy cover (%)	9.67 ± 2.87[c]	29.9 ± 7.20[b]	93.8 ± 1.05[a]	<0.001
Herb biomass (kg/ha)	4008 ±361[a]	733 ± 79[c]	1749±231[b]	<0.001
Tree aboveground biomass (Mg/ha)	2.68± 0.85[b]	6.80±1.84[b]	17.3±2.89[a]	0.003

Means with same letter within the row are not significantly different at 0.05 level of significance. The P values were obtained from the general linear models and linear mixed-effects models described in the main document.

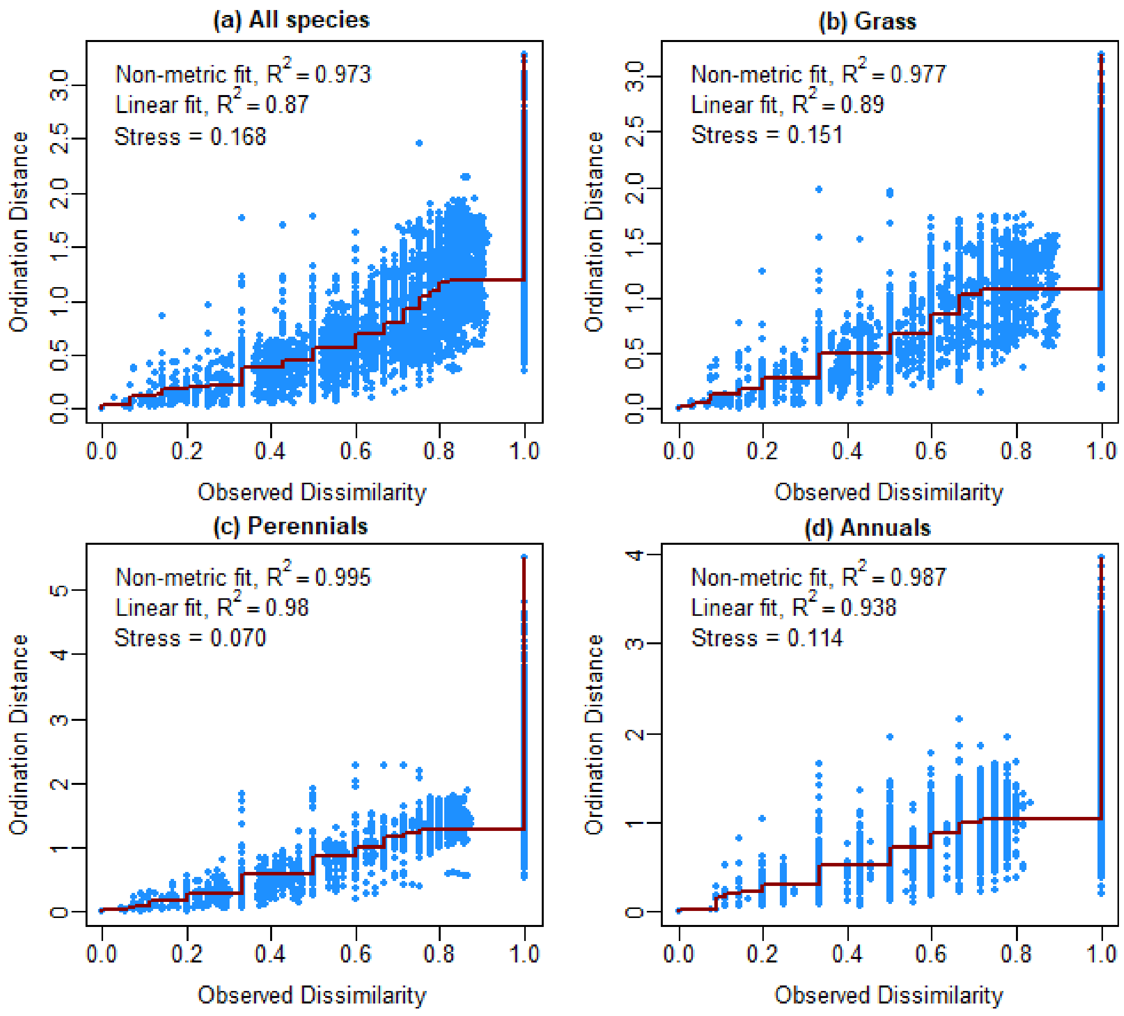

Figure S1. Stress plot of plant community composition from non-metric multidimensional scaling analysis (NMDS) for all species and separately for growth and life forms.
